# How Many proteins are Missed in Quantitative proteomics Based on Ms/Ms sequencing Methods?

**DOI:** 10.4137/PRI.S5882

**Published:** 2010-10-28

**Authors:** Claire Mulvey, Bettina Thur, Mark Crawford, Jasminka Godovac-Zimmermann

**Affiliations:** Division of Medicine, University College London, 5 University Street, London WC1E 6JF, UK

**Keywords:** mass spectrometry, proteomics, MS/MS sequencing

## Abstract

Current bottom-up quantitative proteomics methods based on MS/MS sequencing of peptides are shown to be strongly dependent on sample preparation. Using cytosolic proteins from MCF-7 breast cancer cells, it is shown that protein pre-fractionation based on pI and MW is more effective than pre-fractionation using only MW in increasing the number of observed proteins (947 vs. 704 proteins) and the number of spectral counts per protein. Combination of MS data from the different pre-fractionation methods results in further improvements (1238 proteins). We discuss that at present the main limitation on quantitation by MS/MS sequencing is not MS sensitivity and protein abundance, but rather extensive peptide overlap and limited MS/MS sequencing throughput, and that this favors internally calibrated methods such as SILAC, ICAT or ITRAQ over spectral counting methods in attempts to drastically improve proteome coverage of biological samples.

## Introduction

Current bottom-up quantitative proteomics methods based on MS/MS sequencing of peptides usually only monitor a small proportion of the proteome of higher eukaryotic cells. Typically 2000–3000 proteins are observed,^1-4^ with a maximum so far of 5,111.^1^ Furthermore, the set of observed peptides for any given protein often has limited sequence coverage that makes the recognition of protein isoforms difficult.^5^ Overcoming these limitations is a crucial task for proteomics.

At present it is not entirely clear whether the limited proteome coverage arises from limitations on the sensitivity and sequencing throughput of MS instruments, from inadequate identification of peptides in MS/MS data, from insufficient pre-resolution of the peptides presented to MS/MS sequencing or a combination of these factors.^6^

We present evidence that severe peptide overlap rather than intrinsic MS sensitivity: 1) is a major current bottleneck for much improved proteome coverage, 2) makes the set of observed proteins strongly dependent on sample preparation methods, and 3) can be partially overcome by concurrent use of different, complementary protein/peptide prefractionation methods (here 3D prefractionation based on protein pI, protein MW, peptide HPLC elution time separations). We consider the consequences for common current proteomics strategies, particularly the prospects for dramatically improved proteome coverage using internally calibrated labelling methods such as SILAC, ICAT or ITRAQ^7,8^ versus the use of direct spectral counting methods.^9^

## Methods

The preparation of the MCF-7 breast cancer cells and the fractionation of the cellular proteins according to subcellular organelles by sucrose gradient centrifugation was described in detail previously.^10^ An aliquot of the sucrose gradient fraction corresponding to cytosolic proteins (fraction 9 of ref. 10) was further prefractionated to four pI ranges 3.0–4.6, 4.6–6.2, 6.2–7.0 and 7.0–10.0 using the ZOOM IEF Fractionator from Invitrogen (Paisley, UK) according to the manufacturers instructions. Each protein pI fraction was subsequently subjected to SDS-PAGE gel separation, preparation of tryptic peptides, HPLC separation of peptides and MS/MS analysis as described in detail previously.^10^

## Discussion

We have recently investigated the subcellular distribution of proteins in MCF-7 breast cancer cells using fractionation of subcellular organelles by sucrose density centrifugation.^10^ We noted that as many as 75% of the 2188 proteins identified were consistent with multiple subcellular locations. More than 700 cytosolic proteins were identified, some of which were also observed in other subcellular cellular locations.^10^ This number of cytosolic proteins seemed smaller than expected and raised the question of how many proteins might be missed with current MS/MS sequencing methods.

Based on our previous experiments, we suspected that high abundance proteins were preventing the observation of other proteins, even though in our previous experiments cellular proteins were separated into 24 sucrose gradient fractions and proteins in the cytosolic fraction were further separated by 1D SDS-PAGE gels prior to MS analysis (the experiments are summarized in the footnotes to Table 1). We therefore further fractionated proteins in the cytosolic gradient fraction into four pI ranges (3.0–4.6, 4.6–6.2, 6.2–7.0, 7.0–10.0), each of which was subsequently separated by a 1D SDS gel. This gave a total of 52 samples (4 pI ranges by 13 1D SDS gel slices), which were subjected to HPLC separation and MS/MS sequencing as described in detail previously.^10^ A summary of the assigned MS/MS sequencing events, assigned peptides and assigned proteins is shown in Table 1 (see pI-SDS column), which includes the analogous data for the two repetitions of MS/MS sequencing analysis performed earlier (SDS-1 and SDS-2 columns).

Our previous experiments gave assignments for 539/485 proteins in the SDS-1/SDS-2 data sets respectively. Only 331 of these proteins were common to both experiments, with another 208/154 proteins unique to the SDS-1/SDS-2 experiments. Overall, a total of 704 proteins were identified when the two data sets were combined and the MS data processed jointly (Table 1). This type of result is not uncommon in current quantitative proteomics experiments based on MS/MS sequencing and is often ascribed to incomplete detection of low abundance proteins that are only marginally sampled during the MS analysis. This has often led to the idea that many repetitions of identical experiments are required to better quantify lower abundance proteins, especially in the context of experimental strategies designed to measure changes in cellular function by comparison of different cellular states.

The new experiment (pI-SDS column in Table 1) showed a substantial increase in the number of assigned peptides, which led to assignment of 947 proteins, ie, 243 more proteins than observed previously. Furthermore, joint processing of the MS data for all three experiments led to assignment of 1238 proteins. Comparison of the results of the new experiment with the joint data from the two earlier experiments showed that only 413 proteins were common to both data sets, while 534/291 proteins were uniquely observed in the new/previous data sets (Table 1).

We emphasize that the material used for these analyses all originates from the same preparative sucrose gradient fractionation of the MCF-7 cells and equal amounts of total protein were used for all three MS/MS runs. The magnitude of the changes in the number of assigned proteins therefore strongly suggests that the increases in the number of assigned proteins obtained from repetitions of the same sample preparation method (ie, SDS-1 and SDS-2 here) are *not* a consequence of marginal sampling of low abundance proteins, but instead represent strong masking of peptides by highly abundant peptides as a consequence of current limitations on throughput in MS/MS sequencing experiments.^11^ Thus, the simple expedient of “rearranging” the overlap of peptides by changing the pre-fractionation of the parent proteins resulted in a substantial increase in the number of assigned proteins (Table 1).

**Figure 1:**
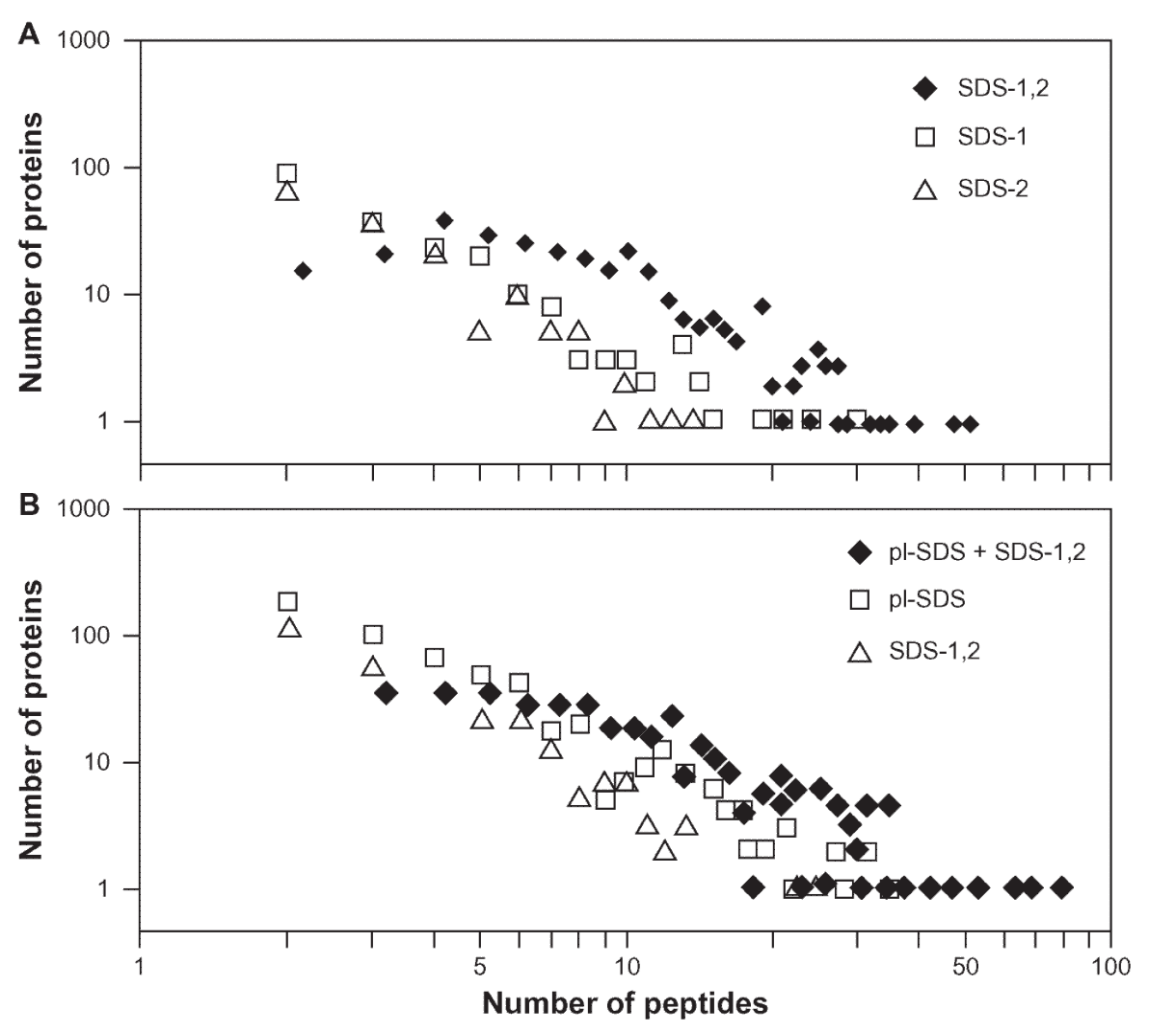
The number of assigned proteins versus the number of assigned peptides contained in the proteins. **A**) Proteins which were observed only in the SDS-1 or SDS-2 data sets, or were common to the SDS-1 and SDS-2 data sets (SDS-1,2). **B**) Proteins which were observed only in the pI-SDS or SDS-1,2 data sets, or were common to the pI-SDS and SDS-1,2 data sets (pI-SDS + SDS-1,2).

To further underpin this conclusion, we looked at the number of peptides that were identified for proteins that were observed jointly or separately between two data sets. Comparing the SDS-1 and SDS-2 data sets showed substantial numbers of proteins for which only a small number of peptides were assigned (Fig. 1). However, for the joint SDS-1,2 data set, 246 (74%) of proteins identified in both individual data sets had ≥5 identified peptides. Interestingly, there are also substantial numbers of proteins with ≥5 identified peptides for proteins observed in only one of the data sets: 60 proteins (29%) for SDS-1 and 33 (21%) for SDS-2. Furthermore, there are several instances where >20 peptides corresponding to a single protein are identified in only one of the two data sets (Fig. 1A). Similar results are obtained by comparing the pI-SDS and SDS-1,2 data sets. For the joint data set, 318 (77%) of proteins identified in both individual data sets had ≥5 identified peptides. 192 (36%) of proteins identified only in the pI-SDS data set and 85 (29%) of proteins identified only in the SDS-1,2 data set had ≥5 identified peptides. There are several instances of proteins with >30 identified peptides that are seen in only one of the two data sets (Fig. 1B). Most of these differences correspond to proteins that gave many peptides, but moderate numbers of total spectral counts.

The detectability of different peptides in proteomics experiments based on MS/MS sequencing is known to vary widely.^12^ However, the present results indicate that even when adequate numbers of peptides with sufficient intrinsic sensitivity are available in a sample, the peptide/protein may not be identified in MS/MS runs corresponding to a specific sample prefractionation. This would seem to be inconsistent with the idea that the sensitivity of the MS spectrometer is the main limiting factor in such experiments.

**Figure 2:**
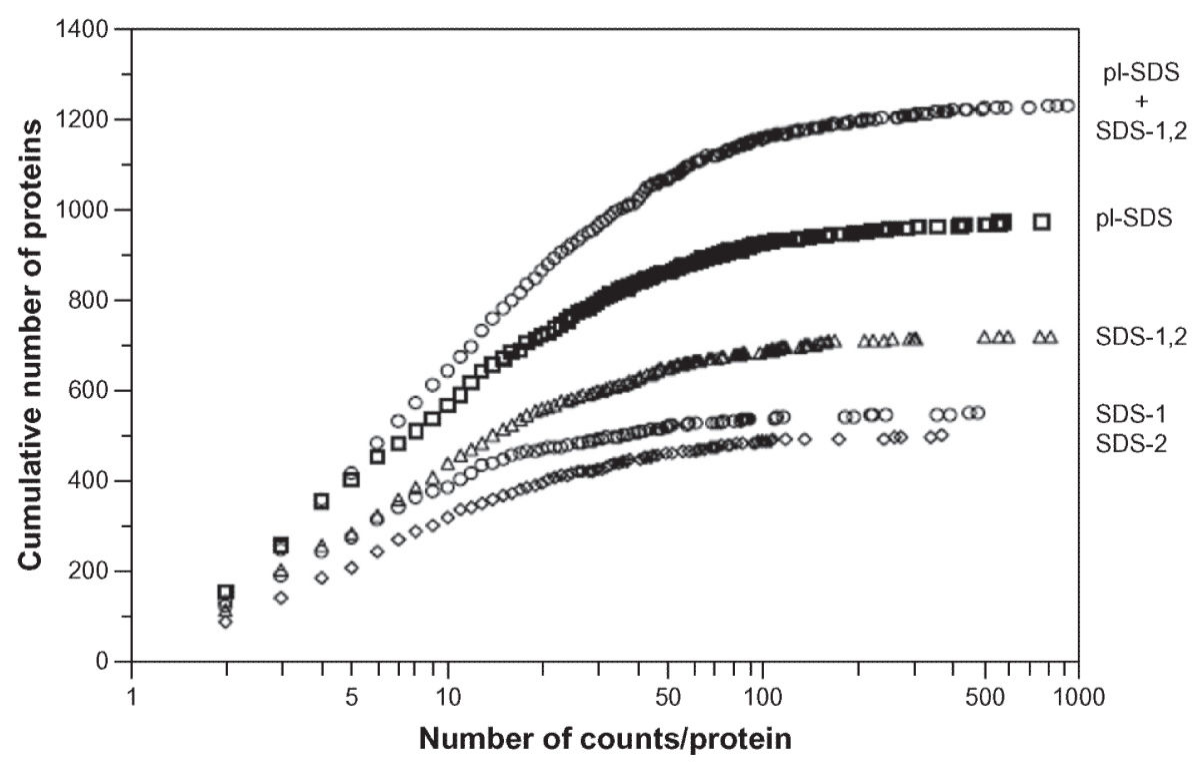
The number of counts per protein versus the cumulative number of proteins with ≤ the number of counts per protein.

We have also looked at how combining MS data from analyses based on different pre-fractionation schemes affects the number of “spectral counts” (assigned MS/MS sequencing events). As shown in Figure 2, in all of the data sets only a small proportion of the proteins had more than 100 counts and most of the assigned proteins had between 2 and 100 counts. For experimental strategies that attempt to quantify changes in protein abundance as a consequence of cellular state, minimum numbers of counts are needed. Interestingly, the pI-SDS data set shows greater numbers of quantifiable proteins than SDS-1,2 (Fig. 2, Table 1), which indicates that a single pre-fractionation based on pI and MW was more effective than repeating the MS measurements with two samples pre-fractionated by MW only. We note that the pI-SDS data set used a total of 52 gel slices whereas the SDS-1,2 data set involved measurements for 42 gel slices, ie, approximately equal amounts of spectrometer time were used. Nonetheless, combination of the two types of data (pI-SDS + SDS-1,2 data set) gave a strong increase in the number of proteins with enough counts for use in relative quantification experiments (Fig. 2, Table 1).

**Figure 3:**
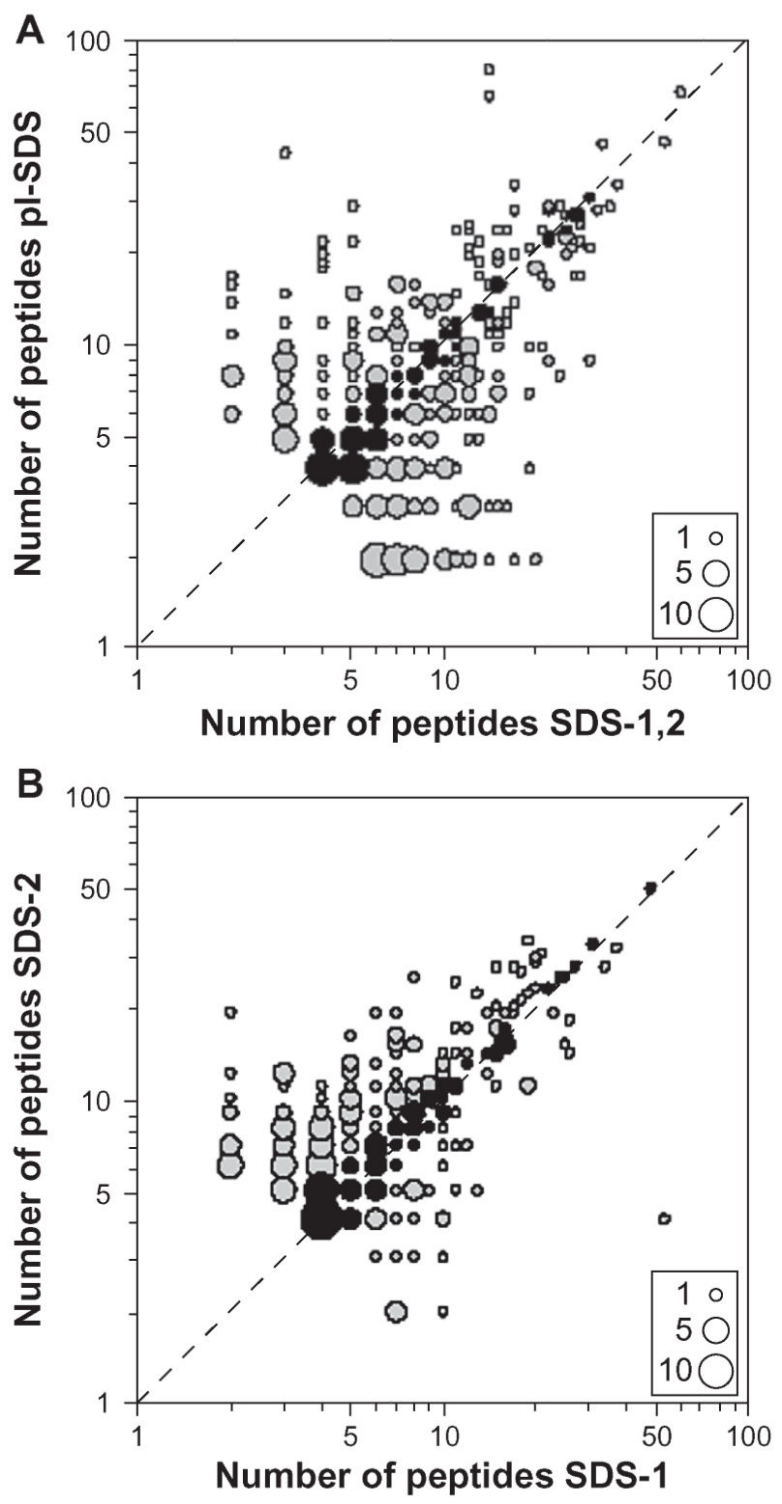
Bubble plots of the number of peptides assigned to individual proteins in two different data sets for proteins with at least 8 assigned peptides over the two data sets. **A**) 185 proteins common to the pI-SDS and SDS-1,2 data sets. **B**) 133 proteins common to the SDS-1 and SDS-2 data sets. The areas of the bubbles (legend) are proportional to the number of proteins showing specific pair combinations of numbers of peptides. Black bubbles shown on or close to the diagonal correspond to differences in numbers of peptides ≤1.

Several considerations indicate that the variability in the observed proteins/counts is not due to differential loss of proteins during sample preparation. First, the sucrose gradient fraction we have used consists of soluble, cytosolic proteins that are less susceptible to sample loss. Second, samples prepared in the same way (SDS-1 and SDS-2) and samples with different pre-fractionation (pI-SDS and SDS-1,2) show similar variability between different analytical runs (Table 1, Fig. 1). As a further test, we have used the set of common proteins with at least 8 observed peptides over the pI-SDS and SDS-1,2 data sets (185 proteins) and over the SDS-1 and SDS-2 data sets (133 proteins). For each protein in each pair of data sets we plotted the data points: (number of peptides observed in data set A, number of peptides observed in data set B). In both cases a relatively small proportion of the common proteins show identical sets of observed peptides between the data sets (Fig. 3). Different slicing of the 1D gels (18 slices for SDS-1, 24 slices for SDS-2 differently located) was sufficient to cause rather large changes in the number of observed peptides for some proteins. Even when similar numbers of peptides were observed, these were often different peptides. One consequence of such variable sequence coverage is that isoforms of the same protein will be difficult to detect and confirm. Overall these results indicate that even for more abundant proteins the intrinsic detectability of peptides is not the main limiting factor, but rather the overlap of different peptides dependent on sample preparation. Unsurprisingly, substantial changes in the number of observed peptides between the two data sets was approximately correlated with substantial changes in the total number of spectral counts for individual proteins.

These results strongly suggest that because of extensive overlap of peptides, limitations on sequencing throughput rather than on spectrometer sensitivity are the main limiting factor in current quantitative proteomics experiments based on MS/MS sequencing. Indeed, since the simple expedient of “rearranging” the input order of the peptides by changing the pre-fractionation of the parent proteins can alter the proteins which are observed, the total spectral counts obtained for given proteins, and the protein sequence coverage, MS/MS throughput seems to be a major hurdle still to be overcome.

Our results contrast with early results obtained for a much simpler organism.^9^ This may reflect the greater numbers/isoform complexity of proteins in higher organisms and/or the strong increases in spectrometer sensitivity that have been achieved in the past few years. Ironically enough, this overlap problem is reminiscent of problems which have always bedevilled proteomics methods based on 2D gels abundant proteins mask the ability to observe less abundant proteins and observation/quantitation of proteins is very sensitive to sample preparation. Solutions which could overcome this limitation and greatly increase the number of proteins detected in such experiments will not be trivial. Our experiments indicate that more extensive pre-fractionation can strongly increase the number of quantifiable proteins. However, even fractionation of the MCF-7 cellular proteins into 24 different sucrose gradient fractions followed by a “low-resolution” 2D gel (4 pI ranges by 13 SDS gel slices) was not sufficient pre-fractionation to ensure that all proteins with adequate intrinsic sensitivity were observed in a single experiment (Table 1). Furthermore, the amount of instrument time required is already a bottleneck in quantitative proteomics based on MS/MS sequencing and this will only be compounded if further subdivision of samples by pre-fractionation is required to improve protein coverage in biological samples. The good news is that there seem to be far more proteins that can be observed/quantified with the sensitivity of current MS spectrometers than are being routinely identified at present.

The present study also suggests that to achieve increased protein coverage for biological samples it may be more productive to combine data sets recorded with completely different pre-fractionation methods rather than to accumulate multiple repetitions of the same experiment (although some replicates are necessary for reliability). At present MS/MS sequencing is mainly used in two contexts in quantitative proteomics experiments aimed at measuring changes in cellular function: a) methods that are based on comparison of biological heavy/light isotope labelling (SILAC)^7^ or of chemical tagging (ICAT, ITRAQ) (8), and b) spectral counting methods that attempt to quantify proteins through the number of MS/MS sequencing events.^13^ These two strategies differ in that because it is “internally calibrated”, a method such as SILAC does not depend on repeating the same pre-fractionation and can readily accommodate data recorded with different protein pre-fractionation schemes. Indeed, the present experiments suggest that completely different fractionation schemes *should be used* in the context of SILAC style experiments. To the extent that the throughput of MS/MS sequencing remains a major limitation and implies extensive, variable sample pre-fractionation to obtain higher protein coverage in biological samples, it may then be that internally calibrated experiments of the SILAC/ICAT/ITRAQ types have greater potential for quantitative proteomics with vastly improved protein coverage.

## Figures and Tables

**Table 1:** Summary of MS/MS assignments.^1^

Data set	pI-SDS^5^ + SDS-1, 2	pI-SDS^3^	SDS-1, 2^4^	SDS-1^2^	SDS-2^2^	
Total counts assigned	44200	26088	18112	8936	9176	
Peptides assigned	8321	6354	4556	3268	3427	
Proteins assigned	1238	947	704	539	485	
*Common*		*413*	*413*	*331*	*331*	*common*
*Only pI-SDS*		*534*		*208*		*only SDS-1*
*Only SDS-1, 2*			*291*		*154*	*only SDS-2*
Counts/protein^6^						
≥5 counts	886 (72%)	626 (66%)	471 (67%)	311 (58%)	319 (66%)	
≥10 counts	632 (51%)	443 (47%)	317 (45%)	175 (32%)	200 (41%)	
≥40 counts	223 (18%)	141 (15%)	101 (14%)	45 (8%)	53 (11%)	

Notes:

1 At least two peptides assigned at 95% confidence level and at least 99.8% confidence in the protein assignment as defined by Scaffold;

2 Proteins from the cytosolic fraction of a sucrose gradient fractionation of subcellular organelles were subjected to 1D SDS gel separation. The gels were sliced into 18 fractions (SDS-1) or 24 fractions (SDS-2). The MS/MS analysis of these samples using an Orbitrap LTQ MS spectrometer has previously been described in detail (10);

3 30 μg of proteins from the same sucrose gradient fraction were separated by pI into four fractions (3.0–4.6, 4.6–6.2, 6.2–7.0, 7.0–10.0) and then by 1D SDS gels with 13 gel slices taken, for a total of 52 fractions. The MS/MS analysis was by the same methods described in detail previously (10);

4 Jointly processed MS data from the SDS-1 and SDS-2 experiments;

5 Jointly processed MS data from the SDS-1, SDS-2 and pI-SDS experiments;

6 The number (percentage) of proteins with counts exceeding the indicated value.
